# Epigenetic regulation of post-stroke cognitive impairment by gut microbiota and their metabolites

**DOI:** 10.3389/fcimb.2026.1774049

**Published:** 2026-06-22

**Authors:** Ying Weng, Wanqi Yang, Yong Lai

**Affiliations:** 1 College of Pharmacy,Dali University, Dali, China; 2 Yunnan Provincial Key Laboratory of Entomological Biopharmaceutical R&D, College of Pharmacy,Dali University, Dali, China; 3 National-Local Joint Engineering Research Center of Entomoceutics, Dali, China

**Keywords:** epigenetics, gut microbiota, metabolites, microbiota-gut-brain axis, post-stroke cognitive impairment

## Abstract

Post-stroke cognitive impairment (PSCI) is a common and disabling complication after stroke, yet its underlying mechanisms remain incompletely understood. Emerging evidence indicates that gut microbiota (GM) and their metabolites play a critical role in the pathogenesis of PSCI through the microbiota-gut-brain (MGB) axis. Increasing studies have demonstrated that GM dysbiosis after stroke leads to alterations in microbial metabolites, including short-chain fatty acids (SCFAs), B vitamins, tryptophan metabolites, bile acids, and other neuroactive compounds, which can influence neuroinflammation, blood–brain barrier integrity, synaptic plasticity, and neuronal survival. Notably, many of these metabolites participate in epigenetic regulation, such as DNA methylation, histone modification, non-coding RNA regulation, chromatin remodeling and RNA modifications, thereby affecting gene expression related to cognitive function and neural repair. This review summarizes recent advances in the relationship between gut microbiota, microbial metabolites, and epigenetic mechanisms in PSCI, and discusses how microbiota-derived metabolites mediate epigenetic reprogramming involved in neuroinflammation, oxidative stress, and neuronal apoptosis. Furthermore, this review highlights potential therapeutic strategies targeting the gut microbiota and their metabolites, including microbiota modulation, metabolite supplementation, and epigenetic intervention. Understanding the interaction between gut microbiota-derived metabolites and epigenetic regulation may provide new insights into the pathogenesis and treatment of PSCI and support the development of personalized therapeutic strategies.

## 1 Introduction

Stroke remains the leading cause of death and disability worldwide, with approximately 13 million new cases reported annually (Daoud et al., 2024). Accumulating evidence indicates that stroke significantly increases the risk of cognitive impairment, with stroke survivors exhibiting a 5-8-fold higher risk compared with the general population (Guo et al., 2024). Post-stroke cognitive impairment (PSCI) is one of the most common neuropsychiatric complications after stroke and may progress to dementia, placing a substantial burden on patients, families, and healthcare systems (Filler et al., 2024). Ischemic stroke (IS), accounting for approximately 75%-85% of all stroke cases, is particularly associated with cognitive decline and various neuropsychiatric disorders (Martí-Carvajal et al., 2020; Gallucci et al., 2024; Zhang et al., 2024; Soni et al., 2025). PSCI is characterized by deficits in memory, attention, executive function, and learning ability, and it significantly reduces quality of life and survival among stroke survivors (Kim et al., 2020; Chi et al., 2023; Luo et al., 2024). Therefore, early identification of PSCI mechanisms and effective interventions is of great clinical importance.

However, impaired learning and memory are recognized as its primary manifestations (Zeng et al., 2024). Among these mechanisms, damage to the central cholinergic system plays an important role in cognitive decline after stroke (Gao et al., 2020). Following ischemic injury, cholinergic neurons are damaged through endoplasmic reticulum stress, oxidative stress, and inflammatory responses, leading to impaired cognitive function (Dai et al., 2025). Due to the overlap between PSCI and Alzheimer’s disease (AD) in cholinergic dysfunction, several AD-related therapeutic strategies, such as cholinesterase inhibitors, have been explored for PSCI treatment (Hampel et al., 2018; Kim et al., 2020; Zhang et al., 2021). However, current treatments for PSCI are still largely derived from general stroke management strategies and remain limited in effectiveness and safety (Wen et al., 2025). These limitations highlight the need to explore new mechanisms and therapeutic targets for PSCI.

In recent years, increasing attention has been paid to the role of gut microbiota (GM) in neurological diseases. GM is now recognized as an important regulator of host metabolism, immune function, and nervous system homeostasis (Yuan et al., 2021; Li et al., 2022; Wang et al., 2022). Under physiological conditions, GM maintains intestinal homeostasis; however, stroke can disrupt gut microbiota composition, while gut microbiota dysbiosis may in turn influence stroke outcomes, forming a bidirectional interaction (Wang et al., 2022; Liu et al., 2024; Yang et al., 2024; Zhang et al., 2024) This bidirectional communication is mainly mediated through the microbiota-gut-brain (MGB) axis, which involves neural, immune, and endocrine pathways (Socała et al., 2021). Importantly, a study has shown that PSCI is closely associated not only with changes in GM composition but also with alterations in microbial metabolites (Liu et al., 2020), These metabolites are increasingly considered potential biomarkers and therapeutic targets for PSCI (Tu and Xia, 2024). Therefore, GM and its metabolites have become an important focus in PSCI research.

In parallel, epigenetic modifications have emerged as key regulators of gene expression and neuroplasticity during post-stroke recovery (Jeong et al., 2023). These modifications, which include DNA methylation, histone modifications, and non-coding RNA regulation, exist in a dynamic equilibrium. Dysregulation of these processes has been implicated in multiple pathological conditions, including PSCI. Notably, gut microbiota-derived metabolites such as short-chain fatty acids (SCFAs) have been shown to influence epigenetic processes, including DNA methylation and histone acetylation, thereby regulating gene expression and neural function (Kaur et al., 2021), These findings suggest that GM may influence PSCI development through epigenetic regulation mediated by microbial metabolites.

Despite increasing evidence linking GM, microbial metabolites, and epigenetic regulation to PSCI, the mechanisms underlying this interaction remain incompletely understood. Therefore, this review aims to summarize current advances in the epigenetic regulation of PSCI mediated by GM and their metabolites, and to discuss potential therapeutic strategies targeting the microbiota-metabolite-epigenetic axis in PSCI.

## 2 The gut-brain connection in PSCI

GM comprising trillions of microbial cells, exerts crucial role in maintaining host physiological homeostasis and regulating metabolic. Dysbiosis of GM has been linked to a variety of disorders, including CNS neuropsychiatric disorders such as AD and PSCI. Of note, the altered composition of the GM and metabolite profiles observed in PSCI patients strongly implicate GM dysbiosis in PSCI pathogenesis. Central to this interplay is the MGB axis, a bidirectional communication network connecting the CNS with the enteric nervous system (ENS). Through this axis, GM-derived signals modulate brain function, behavioral responses, and cognitive processes.

### 2.1 The MGB axis in PSCI

The human body harbors an estimated 10–100 trillion symbiotic microbial cells, collectively termed the human microbiota. This complex microbial consortium comprises bacteria, fungi, and archaea (distinct from parasites, which are typically pathogenic), along with their collective genetic material, forming the human microbiome (Honarpisheh et al., 2022; Lamminpää et al., 2024). Critically, the microbiota modulates the host brain function through immunoregulatory pathways and the biosynthesis of diverse metabolites and neuroactive compounds (Kang et al., 2024). The gastrointestinal tract hosts the densest microbial community, its resident microorganisms and associated genomes are defined as the GM (Honarpisheh et al., 2022; Xiao et al., 2022). Recognized as a second genome, the gut microbiome profoundly contributes to human physiology and metabolism, sharing functional synergies with host systems (Cui et al., 2023). Its key roles encompass regulating neurodevelopment, synaptic plasticity and immune homeostasis (Renga et al., 2024). Under physiological equilibrium, GM maintains a stable symbiotic state that is essential for systemic health. However, perturbations induced by endogenous or exogenous stressors can drive GM dysbiosis, disrupting host metabolic and immune networks (Qian et al., 2023). Such dysregulation has been implicated in gastrointestinal pathologies and extra-intestinal disorders, including CNS-associated neuropsychiatric conditions (e.g., AD, Parkinson’s disease, and depression) (Zhuang et al., 2018; Ji et al., 2021; Kolobaric et al., 2024).

Building upon the foundational understanding of GM dynamics, recent research has increasingly focused on PSCI to elucidate its potential association with GM dysregulation. Extensive evidence indicates that GM imbalance is mechanistically linked to both the pathogenesis and clinical outcomes of IS, mediated through bidirectional crosstalk between the gut and CNS via MGB axis (Haupt et al., 2023). The MGB axis encompasses a complex neural network connecting the CNS with the ENS, facilitating reciprocal interactions. Within this framework, the CNS modulates gastrointestinal functions through multisystem integration of vagal signaling, sympathetic pathways, endocrine regulation, immune activation, and direct GM interactions (Ciancarelli et al., 2024). These regulatory mechanisms collectively influence intestinal motility, epithelial barrier integrity, neurotransmitter/hormone secretion, and immune cell recruitment. Conversely, GM-derived signals reciprocally impact cerebral physiology and cognition through identical pathways. Notably, experimental studies have shown that IS-induced neuroinflammation triggers immune cell infiltration into the brain parenchyma (Zhou et al., 2023). Crucially, GM metabolites can prime peripheral immune cells within the gut, which subsequently migrate to ischemic brain regions to mediate neural injury and repair processes (Liu et al., 2020). This GM-CNS-immune interplay underscores the critical role of GM in shaping post-stroke functional recovery, positioning it as a key investigational target for elucidating PSCI mechanisms.

### 2.2 GM dysbiosis in PSCI

Emerging evidence highlights the critical role of GM alterations in the pathogenesis of PSCI (refer **Table 1**). Clinically, PSCI patients present with distinctive GM profiles characterized by reduced α-diversity, compositional dysbiosis, and metabolite depletion (e.g., diminished SCFAs), alongside elevated *Fusobacterium* abundance compared to non-PSCI controls (Liu et al., 2020). Wang (Wang et al., 2022) further demonstrated that PSCI cohorts display exacerbated peripheral inflammation and elevated *Enterobacteriaceae* levels relative to non-PSCI patients. These findings were validated through fecal microbiota transplantation (FMT) experiments, wherein mice colonized with GM from PSCI donors demonstrated elevated *Enterobacteriaceae* abundance, impaired cognitive performance, intestinal barrier dysfunction, reduced fecal butyrate concentrations and upregulated TLR4 expression.

**Table 1:** Changes in GM and its metabolites in diseases related to cognitive impairment.

Changed gut microbiota	Changed gut microbiota metabolites	Pathological changes	Diseases	Reference
*Fusobacterium*↑, *Oscillibacter*, *Ruminococcus*, *Gemmiger*, *Coprococcus*↓	SCFAs(especially hexanoic acid)↓	/	PSCI	(Liu et al., 2020)
*Enterobacteriaceae*↑	butyrate↓	hippocampal apoptosis, thalamic Aβ deposition	PSCI	(Wang et al., 2022)
*Actinomycetes*↑, *Bifidobacterium*, *Alloscardovia*, and *Alloscardovia omnicolens* of the phylum *Actinomycetes*, *Lactobacillus gasseri* and *Anaerostipes hadrus* of the phylum *Bacillota*↑	/	/	PSCI	(Li et al., 2022)
*Pseudomonadota*↑; *Bacteroidaceae*, *Lachnospiraceae*, and *Veillonellaceae*↑, *Enterobacteriaceae*↓;*Bacteroides*, *Clostridium XIVa*, and *Parabacteroides*↑, *Prevotella* and *Ruminococcus*↓	/	/	PSCI	(Hu et al., 2023)
*Enterococcus*, *Bacteroides*, and *Escherichia-Shigella*↑, *Faecalibacterium*↓	/	/	PSCI	(Huang et al., 2021)
/	5-HTP↓, Indole derivatives↑(indole-3-pyruvic acid, 5-hydroxyindole and indole-2-carboxylic acid), SCFAs↓ (2-methylbutyric acid and isovaleric acid)	/	AD	(Chen et al., 2022)
*Bacillota*↓, *Pseudomonadota*↑, *Gammaproteobacteria*↑, *Aeromonas*↑, *Lachnospiraceae_NK4A136*↓, Turicimonas↑	butyrate↓	microglia overactivation and neuronal apoptosis in the hippocampus	sleep deprivation-induced cognitive impairments	(Li et al., 2024)
*Pseudomonadota↑, Alistipes, Bacteroides, Prevotella and Lactobacillus* ↓;*Staphylococcaceae, Enterococci, Psychrobacter, and Oligella↑*	total SCFAs and acetic acid↓	hippocampal neurons were not arranged orderly, and a large number of neurons were degenerated and necrotic	cognitive impairment	(Deng et al., 2022)

↑ indicates an increase, ↓ indicates a decrease.

Moreover, Actinobacterota phylum (e.g., *Bifidobacterium* and *Alloscardovia*) and specific *Bacillota* species (e.g., *Anaerostipes hadrus* and *Lactobacillus gasseri*) were significantly enriched in PSCI patients compared to non-PSCI groups (Li et al., 2022). A meta-analysis corroborated phylum-level dysbiosis, revealing increased *Pseudomonadota* and *Bacteroidaceae/Lachnospiraceae/Veillonellaceae* at the family level in PSCI, though *Enterobacteriaceae* depletion contrasted with Wang’s observations (Hu et al., 2023). This discrepancy underlines the necessity for longitudinal studies clarify the dynamics of *Enterobacteriaceae* across PSCI stages. At the genus level, PSCI patients exhibited elevated *Bacteroides*, *Clostridium XIVa*, and *Parabacteroides*, alongside reduced *Blautia*, *Prevotella*, and *Ruminococcus (*Huang et al., 2021). Notably, *Bacteroidota* enrichment was proposed as a potential PSCI biomarker (Pluta and Januszewski, 2023). Supporting this, 16S rRNA sequencing revealed higher *Enterococcus*, *Bacteroides*, and *Escherichia-Shigella*, but diminished *Faecalibacterium* in PSCI relative to stroke-only cohorts (Huang et al., 2021). Collectively, GM dysbiosis is mechanistically intertwined with PSCI pathogenesis. Future research should focus on the mechanism of GM-host interactions in PSCI and explore the therapeutic strategies via GM modulation (e.g., probiotics, FMT).

### 2.3 The role of microbial metabolites in PSCI pathogenesis

Another significant truth is that GM changes lead to alterations in the production of its metabolites. These metabolites play a crucial role in the functioning of GM. Although the relationship between GM metabolites and PSCI has not been extensively studied, other research on diseases associated with cognitive impairment has indicated that GM metabolites play a significant role in PSCI. As is widely recognized, post-stroke neurodegeneration is multifactorial and characterized by neuroinflammatory responses, neuronal death, amyloid plaques, neuroprogenitor fibre tangles, acetylcholine deficiency, and cognitive deficits with full-blown dementia (Pluta and Januszewski, 2023), which are core pathological manifestations associated with cognitively impaired diseases and even dementia. It is noteworthy that both PSCI and AD are secondary to neuronal cell death due to uncontrolled inflammatory responses, which lead to structural brain damage and resulting in cognitive deficits (Cho et al., 2021). Neuroinflammation is particularly important in the progression of PSCI. Moreover, GM metabolites are involved in processes such as neuroinflammation and oxidative stress in PSCI. Neuroinflammation within the brain is triggered by the release of inflammatory mediators from immune cells like microglia and astrocytes. The process unfolds as follows: when GM homeostasis is disturbed, the integrity of the blood-brain barrier (BBB) is disrupted and metabolites such as endotoxins penetrate the brain, thereby stimulating microglia activation and triggering neuroinflammation. However, tryptophan (TRP) metabolites can also cross the BBB. They modulate the immunoreactivity among astrocytes and microglia, while influencing neuroinflammation centrally. Butyrate can also exert anti-inflammatory and immunomodulatory effects by maintaining the gut barrier or in a variety of other ways (Chen et al., 2022; Li et al., 2024). In addition, oxidative metabolism is essential for neuronal survival, but excessive accumulation of free radicals can lead to oxidative stress. This oxidative stress causes cellular dysfunction, apoptosis, necrosis and ultimately cell death, and ultimately memory impairment (Deng et al., 2022; Xia et al., 2023). Dysbiosis further exacerbates oxidative stress. Additionally, the increased production of reactive oxygen species contributes to neuroinflammation. Metabolites of GM such as butyric acid and vitamin B modulate the oxidative state of the CNS and play a role in preventing oxidative stress and inflammation (Shabbir et al., 2021). A high-throughput UPLC/MS and GC/MS-based fecal metabolomics has identified a fecal microbiological signature of AD, pointing to fecal metabolic dysregulation in AD patients, pointing to TRP, SCFA and bile acid pathways, which have been linked to GM disorders and cognitive impairment (Wu et al., 2021). An experimental study on animals demonstrated that the transplantation of GM from sleep-deprived mice with cognitive deficiencies into normal mice resulted in the activation of microglia in the hippocampus and neuronal apoptosis, cognitive decline, and disruption of the colonic microbiota. This was evidenced by elevated levels of *Aeromonas* and lipopolysaccharide (LPS) along with reduced levels of *Lachnospiraceae_NK4A136* and butyrate. Furthermore, the administration of SCFA (butyrate) to sleep-deprived mice led to the restoration of inflammatory responses and memory impairment (Wang et al., 2023b). In accordance with this, Xiang (Lu et al., 2022) demonstrated that fluctuations in GM and its metabolites are responsible for cognitive deficits observed in preterm rats. Evidence included a substantial decrease both in GM abundance and diversity coupled with a significant increase in *Pseudomonadota* abundance in rats within the cognitively impaired group compared to normal control rats. Furthermore, a significant reduction in the total concentrations of SCFA and acetic acid was observed in the faeces of rats exhibiting cognitive impairment. It is noteworthy that GM-derived metabolites appear to play a pivotal role in various causes of cognitive impairment, underscoring the necessity to study GM and its metabolites in relation to PSCI. Differently, specific varieties of SCFAs vary in cognitive impairment due to different diseases. Experiments are needed to further reveal the specific SCFAs that affect PSCI. Recent studies have indicated that GM can modulate the epigenetic landscape of the host through bacterial components and microbial metabolites. Butyrate, a constituent of SCFAs, has been identified as a potent inhibitor of histone deacetylase. In addition, other bacterial metabolites, including folate, choline, and trimethylamine-N-oxide, have been observed to modulate epigenetic mechanisms (Begum et al., 2022). GM metabolites have been demonstrated to modulate a variety of diseases by inducing epigenetic alterations through DNA methylation, histone modification, and the silencing of non-coding RNA-related genes. Pharmacological treatment with butyrate has been found to potentially create an enriched epigenetic platform in the hippocampus and to improve stroke-induced cognitive dysfunction (Okamura et al., 2023). Consequently, the objective of this study was to elucidate a correlation between GM metabolites and epigenetic modifications, proposing them as potential targets for the diagnosis and treatment of PSCI.

## 3 Gut microbiota–host crosstalk modulates epigenetic mechanisms in post-stroke cognitive impairment

Epigenetics is the study of heritable alterations in gene expression that are independent of major changes in the DNA sequence and depend on the interaction between environmental factors and the genome (Phillips et al., 2023). Epigenetic modifications mainly include covalent modifications of DNA (methylation) and post-translational modifications of histone N-terminal tails (acetylation, methylation, phosphorylation, and ubiquitination, among others), as well as non-coding RNA regulation (Jin et al., 2017). There is mounting evidence that epigenetic modifications have emerged as significant contributors to recovery following stroke. Firstly, DNA methylation and demethylation are mediated by DNA methyltransferases (DNMTs) and 10–11 translocation proteins, which ultimately lead to gene silencing and expression (Liu et al., 2020). DNA methylation affects neuroplasticity and cognitive function by regulating gene expression. Alterations in DNA methylation levels of specific genes have been observed in patients with PSCI, suggesting a potential role for these modifications in the pathological progression of the condition. Secondly, all histone modifications are catalyzed by histone acetyltransferase (HAT) and histone deacetylase (HDAC) (Rroji and Mucignat, 2024). Post-translational modifications of histones ultimately lead to transcriptional activation or repression (Stamatovic et al., 2019). GM metabolites like short-chain fatty acids have been shown to influence histone modifications through inhibiting HDAC, thereby affecting histone modifications and potentially ameliorating the neuroinflammation and synaptic plasticity associated with PSCI. Finally, non-coding RNAs (particularly microRNAs) play crucial role in post-transcriptional regulation of gene expression. They serve as powerful regulators of various cellular activities, including cell growth, differentiation, development and apoptosis (Saliminejad et al., 2019). Non-coding RNAs modulate gene expression and may alleviate the pathological manifestations associated with PSCI by reducing apoptotic neurons and attenuating neuroinflammation. The above epigenetic modifications play key roles in regulating gene silencing and expression, and these modifications are important in the development and progression of PSCI. It has recently been established that GM and its metabolites significantly contribute to the development of the host’s epigenetic landscape in the context of PSCI. Furthermore, environmental signals derived from the gut microbiome have been shown to influence the host’s response to stimuli by means of modifying the host epigenome. This underscores the necessity for additional research in this domain to elucidate the mechanisms through which GM exerts its effect on the cognitive levels of PSCI by modulating epigenetic modifications.

### 3.1 DNA methylation in PSCI

It is evident that DNA methylation, histone post-translational modifications, and non-coding RNA regulation play pivotal roles in gene silencing and expression, and that these modifications are instrumental in the development and progression of PSCI. Previous study has demonstrated that DNA hypermethylation is associated with cognitive, spatial and social memory deficits induced by cerebral ischaemia/reperfusion in rats, and that DNA hypermethylation in the *Ngf* gene leads to cognitive deficits after stroke (Fan et al., 2023). However, hypomethylation of specific genes has also been linked to cognitive impairments in IS and early post-onset, including the hypomethylation of the *RIN3* gene (Miao et al., 2021). Furthermore, a study have shown that reducing the expression of DNMT3A, DNMT3B, and DNA methylation of CpG islands in the promoter of the brain-derived neurotrophic factor (BDNF) gene in the mouse hippocampus results in increased expression of BDNF and activation of BDNF/TrkB signaling, which ultimately facilitates learning and memory. BDNF, a member of the neurotrophic factor family, is highly expressed in the hippocampus of the brain and is responsible for synaptic plasticity in the CNS, neurotransmitter release and neuronal development in the CNS (Li et al., 2021; Zhang et al., 2023). A number of studies have demonstrated that GM can influence DNA methylation in various diseases (Ansari et al., 2020; Zhang et al., 2023), as found by Mariella Cuomo (Cuomo et al., 2021). In the gut and brain of zebrafish, *L. rhamnosus* leads to a remodeling of the DNA methylation code of BDNF and Tph1A promoter genes. In addition, neuroinflammation is the primary cause of neurological damage as well as spatial and memory decline in PSCI (Zhang et al., 2021b). GM-dependent adapter molecules recruit DNA methyltransferase to the TLR4 gene in colonic epithelial cells, thereby inhibiting the inflammatory response (Narabayashi et al., 2022), and that inflammatory states in the gut can be transmitted to the CNS via the ‘gut-brain axis’ (Zhao et al., 2021). It is hypothesized that GM can inhibit the inflammatory cascade and thereby protect neurons through epigenetic modifications; however, further studies are needed to justify this hypothesis. In short, DNA methylation can exert a pivotal influence on the pathogenesis of PSCI by regulating the expression of cognitively related genes, and GM also appears to hold a significant position in this complex process. Indeed, GM may assume a central function in the inhibition of neuroinflammation and neuronal protection in PSCI by regulating DNA methylation. This epigenetic regulatory mechanism offers a profound insight into the molecular interactions of the MGB axis and provides a theoretical foundation for the development of cognitive protection strategies based on colony intervention.

### 3.2 Histone post-translational modification

In a similar manner, histone post-translational modification represents a significant epigenetic regulatory mechanism. Histone acetylation has been demonstrated to be closely associated with learning and memory processes within the brain (Bergamasco et al., 2024). The process of histone acetylation is facilitated by HAT and HDAC, with SCFAs produced by GM, notably butyric acid, recognized as HDAC inhibitors (Yuille et al., 2018; Wang et al., 2023a). It has been demonstrated that HDAC is significantly elevated in the hippocampal region of rats in the cerebral ischemia model, which reduces the acetylation levels of histones H3 and H4 in rats. Treatment with sodium butyrate has been shown to enhance the acetylation levels of histone H3 and H4 by inhibiting HDAC, as well as to elevate the levels of BDNF in brain tissues, thereby improving the cognitive deficits of stroke rats (Gao et al., 2021). This finding is consistent with a study by Tu (Tu et al., 2017). In addition, histone methylation also exerts a critical influence on pathological manifestations related to cognition. It is further noted that GM metabolites can provide methyl donors for methylation reactions, a subject to be addressed in the fourth part of the article. Emerging evidence further indicates that histone lactylation exerts substantial regulatory impacts on neurodegenerative diseases by modulating glial function and neuroinflammation (Wang et al., 2024).

It has been demonstrated that the transcriptional activator GATA-binding protein 3 attenuates transient middle cerebral artery occlusion by recruiting the epigenetic regulator lysine-methyltransferase-2A, which increases the trimesterated lysine-4 of histone-3 (H3K4-3me), resulting in an increase of NCX3 expression in brain induced injury (Guida et al., 2021). The regulation of histone H3 lysine 4 (H3K4) methylation has been linked to memory formation in the mouse hippocampus and mutations in several genes encoding H3K4 methylation modifiers have also been linked to cognitive dysfunction in humans (Collins et al., 2019). Emerging evidence suggests that histone lactylation may regulate glial function and neuroinflammation in neurodegenerative diseases (Wang et al., 2024). Acetylation, methylation and lactylation of histone proteins are closely related to the pathological process of PSCI, and they exert significant effects on PSCI by regulating the expression of relevant genes or participating in the pathological processes of apoptosis, glial function and neuroinflammation associated with PSCI. Importantly, GM-derived SCFA provides a novel target for intervention to reshape the histone acetylation landscape by inhibiting HDAC activity. Future studies should focus on the effect of GM on other histone modifications, to explore the interactions between different modification types and how colony metabolites spatiotemporally and temporally specifically modulate the histone modification landscape involved in the pathogenesis of PSCI.

### 3.3 Non-coding RNA regulation in PSCI

Non-coding RNA regulation is an emerging epigenetic mechanism after DNA methylation and histone modification, especially microRNA (miRNA), which has emerged as a critical modulator of various diseases and contributes significantly to PSCI pathogenesis. For example, after establishing a mouse model of cerebral ischemia using middle cerebral artery occlusion, miRNA-532-5p expression levels were significantly reduced, enabling its overexpression to attenuate neuronal damage and apoptosis and prevent IS by inhibiting *PTEN* and activating the PI3K/Akt signaling pathway (Mu et al., 2020). In addition, high expression of miRNA-135b-5p can alleviate neurological deficits and neuronal injury after stroke and reduce neuronal apoptosis and inflammatory response following cerebral ischemia/reperfusion, which are beneficial for the treatment of PSCI (Huang et al., 2022). Furthermore, it has been suggested that miRNA-511-3p may be involved in the development of stroke and could serve as a novel biomarker to predict the occurrence of PSCI in stroke patients (Wang et al., 2024). A study on sepsis showed that several miRNAs on intestinal epithelial cells of diseased mice may have the potential to regulate anti-inflammatory and pro-inflammatory responses by post-transcriptional modification of their functional target genes (Caidengbate et al., 2023). This finding provides valuable insight into the basis of miRNAs as novel markers for predicting PSCI, elucidate miRNA-DNA crosstalk in PSCI, and reveal whether these miRNAs modify target genes in PSCI through post-transcriptional modification via epigenetic mechanisms. Moreover, it has been reported that dysregulated miRNAs in the cerebral cortex of germ-free mice may be influenced by the GM (Zhang et al., 2021a), and that gram-negative *Bacteroides fragilis* (species) prevalent in the gut activates neuroinflammatory pathways and innate immunity by inducing miRNA-146a and stimulating NF-κB (Ayyanar and Vijayan, 2025). The absence of research in this area thus far presents both opportunities and inspiration. The prospect of comprehending host epigenetic interactions between GM and PSCI through the mechanism of non-coding RNA is both encouraging and stimulating.

GM plays a crucial role in regulating the host epigenetic landscape and influencing disease progression in PSCI, which is achieved either through direct interactions with host cells or through the production of metabolites. In summary, understanding the complex interactions between GM and host epigenetic modifications is critical for the development of new therapeutic strategies for PSCI. Further studies are necessary to elucidate the specific mechanisms by which GM regulates the host epigenetic landscape, including DNA methylation, histone modifications and non-coding RNA-mediated regulation. The acquisition of this knowledge may facilitate the development of targeted therapies and personalized medical approaches for the management and treatment of PSCI, taking into account the composition of the individual GM and epigenetic profiles.

### 3.4 Chromatin remodeling and RNA modifications in PSCI

Chromatin remodeling and RNA modifications represent crucial layers of epigenetic regulation beyond the well-studied mechanisms of DNA methylation, histone modification, and non-coding RNA regulation. These processes tightly control gene expression by altering chromatin accessibility, as well as RNA stability, translation, and degradation, and may play significant roles in the pathogenesis of PSCI (Nossent, 2023; Nagao et al., 2025).

Chromatin remodeling involves the ATP-dependent restructuring of chromatin architecture, which changes nucleosome positioning and chromatin accessibility to regulate transcriptional activity (Wu, 1997). Chromatin remodeling complexes, including SWI/SNF, ISWI, and CHD complexes (Clapier and Cairns, 2009), are fundamental for neurogenesis and neuronal development (Gong et al., 2021b; Jiang et al., 2023; Chmykhalo et al., 2025). Dysregulation of these complexes has been implicated in learning and memory deficits, as well as various neurodegenerative diseases (Kim and Kaang, 2017). After ischemic stroke, inflammatory signaling and oxidative stress can disrupt chromatin structure and the transcription of genes associated with neuronal survival, inflammation, and synaptic plasticity (Zhao et al., 2016). Furthermore, emerging evidence highlights the influence of gut microbiota and their metabolites on chromatin remodeling. These microbiota-driven effects occur through mechanisms, such as modulating histone modifications, DNA methylation, and chromatin accessibility (Bhat and Kapila, 2017; Woo and Alenghat, 2022), thereby indirectly regulating gene expression involved in neuroinflammation and neuronal repair (Pan et al., 2014; Pavlou et al., 2023).

Similarly, RNA modifications, particularly N6-methyladenosine (m6A), have recently emerged as pivotal epigenetic regulatory mechanisms in neurological diseases (Zhang et al., 2026). m6A modification is dynamically regulated by methyltransferases (“writers”), demethylases (“erasers”), and RNA-binding proteins (“readers”), which collectively influence RNA stability, splicing, translation, and degradation (Feng et al., 2023). Increasing evidence indicates that m6A modifications play vital roles in learning and memory, with modulation of RNA methylation shown to improve hippocampus-dependent cognitive function (Du et al., 2021). Notably, recent research suggests that gut microbiota can significantly impact mRNA expression levels in the hippocampus, further supporting a connection between microbial activity and RNA regulation (Chen et al., 2017).

In summary, chromatin remodeling and RNA modifications provide additional layers of epigenetic regulation that may serve as key mediators linking gut microbiota, microbial metabolites, and PSCI. Future research should delve deeper into the interaction between chromatin remodeling complexes and RNA modifications within the microbiota–metabolite–epigenetic regulatory network, advancing our understanding of their roles in PSCI and opening avenues for potential therapeutic strategies.

## 4 GM metabolites mediate host epigenetic reprogramming in PSCI

Many of the GM-mediated CNS effects depend on hundreds of metabolites and bioactive molecules that may enter the systemic circulation, reach the brain and affect the function of large parts of the neural population, including neurons, microglia and astrocytes (Cuartero et al., 2024). This is significant for understanding the pathogenesis of PSCI. For instance, metabolites such as γ-aminobutyric acid, TRP and SCFAs can affect cognitive functions and behaviors in the brain through direct or indirect effects (Peh et al., 2022). Among them, GM converts TRP to 5 -hydroxytryptamine (5 +-HT), which can lead to cognitive changes such as impaired long-term memory formation (Riedel et al., 1999). Butyrate improves functional outcome in aged stroke mice by inducing vascular remodeling and increasing the integrity of the BBB (Chen et al., 2023). Another study indicated that bile acids have a protective effect on the prognosis of patients with clinical IS by promoting the recovery of neurological function through the gut-brain axis (Wang et al., 2024). Preclinical studies reviewed suggest that vitamin B12 may improve behavioral outcomes, including reducing cognitive deficits after traumatic brain injury, by reducing apoptosis due to endoplasmic reticulum stress (Joshi et al., 2024). Importantly, changes in these metabolites have been linked to epigenetic alterations in the pathogenesis of PSCI (Chen et al., 2022; Wang et al., 2023; Zhou et al., 2023). The above findings highlight the crucial role of GM in regulating CNS function. Moreover, the fact that GM regulates epigenetic modifications through its metabolites may contribute to the amelioration or treatment of PSCI, providing a scientific basis for the development of new therapeutic strategies (refer **Table 2**).

**Table 2:** PSCI-related epigenetic mechanisms affected by gut microbiota metabolites.

Gut microbiota	Gut microbiota metabolites	Epigenetic mechanism		Pathological changes	Disease	Reference
/	/	methylation	hypermethylation in *Ngf* gene	The neuronal spines decrease in the cortex	cognitive deficits post-stroke	(Okamura et al., 2023)
/	/		hypomethylation of the *RIN3* gene	/	ischemic stroke with early cognitive impairment	(Liu et al., 2020)
/	/		hypermethylation in *BDNF* gene; DNMT3A, DNMT3B↑	/	hypoxia/ischemia diseases-induced Cognitive dysfunction	(Rroji and Mucignat, 2024)
/	folic acid		DNA methylation on glucocorticoid receptor	Hippocampal Neurogenesis is promoted.	folic acid deficiency	(Uebanso et al., 2020)
	Vitamin B12		m6A hypermethylation of *Prkca* mRNA	/	neurological disease(such as cognitive dysfunction, mental retardation, or memory impairment)	(An et al., 2024)
/	/	histone modification	acetylated H3 and H4 downregulated	hippocampal neuronal damage	cognitive deficits post-cerebral ischemic stroke	(Narabayashi et al., 2022)
/	/		acetylated H3 and H4 downregulated	The hippocampal neurons were arranged loosely	Post-Stroke Cognitive Deficits	(Zhao et al., 2021)
Faecalibaculum ↓, Ruminiclostridium-1↓	SCFAs (butyric acid)↓		acetylation of GSK3β at lysine 15 regulated its phosphorylation at serine 9	Tau phosphorylation, Aβ accumulation	AD	(Marsiglia et al., 2023)
/	/	non-coding RNA regulation	miRNA-532-5p downregulated	neuronal apoptosis	ischemic stroke	(Gao et al., 2021)
/	/		miRNA-511-3p downregulated	/	PSCI	(Tu et al., 2017)

### 4.1 SCFAs

SCFAs are bioactive lipids that serve as metabolites released in the colon following bacterial fermentation of dietary fibre (Hart et al., 2024). They primarily consist of acetic acid, propionic acid and butyric acid, exerting critical regulatory functions in the MGB axis (Gao-Qiang et al., 2023). SCFAs are mainly produced in the colon via monocarboxylate transporter (MCT1) and sodium-dependent monocarboxylate transporter (SMCT1) mediate active transport for rapid uptake by colonic cells (Xue et al., 2023). Importantly, SCFAs can cross the BBB to reach the brain, which may be due to the high expression of MCTs on endothelial cells in the relative order of butyrate, propionate and acetate (Chen et al., 2024). Consequently, microbiota-derived SCFAs can transmit information from the gut to the brain via the MGB axis, thereby serving as critical mediators in regulating brain function through multiple pathways.

A large body of research suggests that patients have abnormal levels of SCFAs after stroke onset (Henry et al., 2021; Chou et al., 2023; Marsiglia et al., 2023), and deficiency of SCFAs have been observed particularly in patients with PSCI (Tan et al., 2021). Previous studies have shown that GM and SCFAs are potential therapeutic targets for IS, and that SCFAs may modulate neuronal plasticity through immune mechanisms, thus contributing to the recovery process after stroke. Transplantation of fecal microbiota enriched in SCFAs and butyric acid has been shown to be an effective treatment for IS (Chen et al., 2019; Sadler et al., 2020). Similarly, transplantation of AD mice feces into wild-type mice resulted in symptoms of cognitive impairment in the wild-type mice. Administration of *Lactobacillus* (genus) and *Bifidobacterium* (genus) to wild-type mice may have modulated the phosphorylation of GSK3β at lysine 15 through the production of butyric acid, thereby mediating its acetylation at serine 9. This modulation further reduces tau phosphorylation and attenuating cognitive deficits in wild-type mice (Zhang et al., 2023). However, there remains uncertainty as to whether supplementation with SCFAs exerts a beneficial effect on stroke-induced cognitive deficits, and the study by Guo (Guo et al., 2023) provided an answer to this question. They showed in animal experiments that the levels of SCFAs in the intestine of rats treated with Chinese herbs for IS were significantly increased, and these intestinal SCFAs were translocated to the brain, where they inhibited neuronal apoptosis and attenuated neuroinflammatory responses. This series of changes led to a significant reduction in brain neuropathy and oxidative stress levels in stroke rats, which in turn ameliorated cerebral ischemia-induced cognitive dysfunction (Guo et al., 2023). This suggests that SCFAs play an important neuroprotective role in PSCI, and that future treatments for PSCI could be administered by modulating GM or directly supplementing SCFAs to significantly ameliorate neuroinflammation, oxidative stress and neuronal apoptosis after stroke, thereby alleviating cognitive dysfunction. To promote these therapeutic modalities in the clinic, it should be further elucidated that the process of interaction between SCFAs and the pathogenesis of PSCI, and reveal the mechanism by which SCFAs act on PSCI.

SCFAs can alter gene expression patterns by inhibiting HDACs, modulating DNA methylation and influencing the expression of non-coding RNAs, and these processes, which together act on aspects of neuroinflammation and synaptic damage associated with PSCI, may be one of the potential mechanisms by which SCFAs act. This is because sodium butyrate, as an HDAC class I and II inhibitor, can induce general chromatin accessibility by upregulating histone acetylation levels, which in turn selectively inhibits pro-inflammatory gene expression and exerts a neuroprotective effect on IS (Patnala et al., 2017). In neurons *in vitro* or *in vivo*, class I HDAC inhibitors enhance neurite outgrowth, synaptic plasticity, neurogenesis, dendritic arborisation and axon regeneration (Takamatsu et al., 2017). These effects have been demonstrated in a variety of neuropsychiatric disorders with cognitive decline (Chen et al., 2022; Javani et al., 2023; Torres-López et al., 2023; Zhao et al., 2023; Wu et al., 2024). Although the majority of research on sodium butyrate has focused on its effects on histone acetylation, some studies indicate that it also affects DNA methylation processes (Wang et al., 2024). For instance, butyrate prevents α-synuclein-induced DNA damage by upregulating DNA repair genes in a mouse model of Parkinson’s disease (Chung and Kim, 2024). Synucleinopathies exhibit several common features in terms of clinical presentation and pathological changes, and clinically manifest as chronic and progressive motor, cognitive, behavioral and autonomic decline (Yang and Yu, 2017). This finding offers a novel perspective on our comprehension of how SCFAs mediate the pathogenesis of PSCI by regulating DNA methylation modifications. In addition, SCFAs and protocatechuic acid produced by GM through the metabolism of certain phytochemicals can inhibit the activity of DNMT1 and DNMT3B (Huang et al., 2020). Notably, dynamic DNA methylation catalysed by DNMTs is involved in CNS synaptic plasticity and cognitive memory formation (Wang et al., 2022). There is also evidence that SCFAs can affect non-coding RNA levels in the host, and that probiotic strains generating SCFAs and lactate modulate the expression of miRNAs, resulting in anti-inflammatory effects and promoting the gut barrier (Kopczyńska and Kowalczyk, 2024), which would reduce the transmission of inflammatory factors to the CNS via the ‘gut-brain axis’. Thus, SCFAs are potential targets for the future treatment of PSCI by affecting histone acetylation and DNA methylation. SCFAs can ameliorate the pathological manifestations associated with PSCI, such as synaptic plasticity and neuroinflammation (Li et al., 2023). However, current studies focusing on the interaction process between SCFAs and epigenetic modification to reveal the pathogenesis of PSCI are still relatively scarce. This presents new opportunities and challenges, and this area may emerge as a focus of attention and a research hotspot in the future.

### 4.2 B vitamins: epigenetic mechanisms and translational challenges in PSCI

Vitamin B can be synthesized by GM or provided by the diet. With the aid of specific transporters, dietary vitamin B is mainly absorbed in the small intestine, whereas GM synthesized vitamin B is absorbed in the colon, where the bacterial flora is dense (Wan et al., 2022). B vitamins in the distal colon may have several important functions *in vivo*, including as modulators of immune cell activity, nutrients for the host and its microbiota, and mediators of drug efficacy (Uebanso et al., 2020). Vitamin B has been shown to exert significant effects on maintaining neurological function in the brain, with vitamin B12, vitamin B9 (folic acid) and vitamin B6 affecting cognitive function (An et al., 2024). A cross-sectional study showed that low blood folate levels are an important cause of cognitive impairment in stroke survivors (Pascoe and Linden, 2016). Sandvig (Sandvig et al., 2024) carried out the first study of biomarkers of vitamin B6 status in PSCI and encouraged further research into the potential preventive effects of vitamin B6 supplementation in PSCI. Additionally, other study has found that low vitamin B12 level is associated with cognitive decline and AD (Yahn et al., 2021). So how is vitamin B involved in regulating cognitive function? In reviewing the literature, methylation is the most common form of vitamin B involved in epigenetic modification. If methylation is not carried out efficiently, it leads to elevated homocysteine levels, which may be associated with neurodegenerative diseases such as dementia, AD and Parkinson’s disease, and an adequate intake of folic acid is essential to ensure efficient methylation (Mikkelsen et al., 2024). This is because nearly all B vitamins are involved in one-carbon metabolism, which plays a central role in the production of methyl donors in the form of S-adenosylmethionine (SAM), which is the only methyl donor utilized by DNA, RNA, histones and protein methyltransferases (Lyon et al., 2020). Moreover, folate as a core nutrient in the one-carbon metabolic network, can be a limiting factor for DNA methylation (Caffrey et al., 2023). Also, vitamin B can influence pathological processes associated with the development of PSCI through methylated epigenetic modifications. Relevant studies have indicated that maternal folic acid supplementation during pregnancy and lactation alters promoter methylation and expression of glucocorticoid receptor exon 1 mRNA variants in the hippocampus of offspring mice, which ultimately promotes hippocampal neurogenesis and improves learning and memory behaviors in the mouse offspring (Yang et al., 2019).

**Figure 1:**
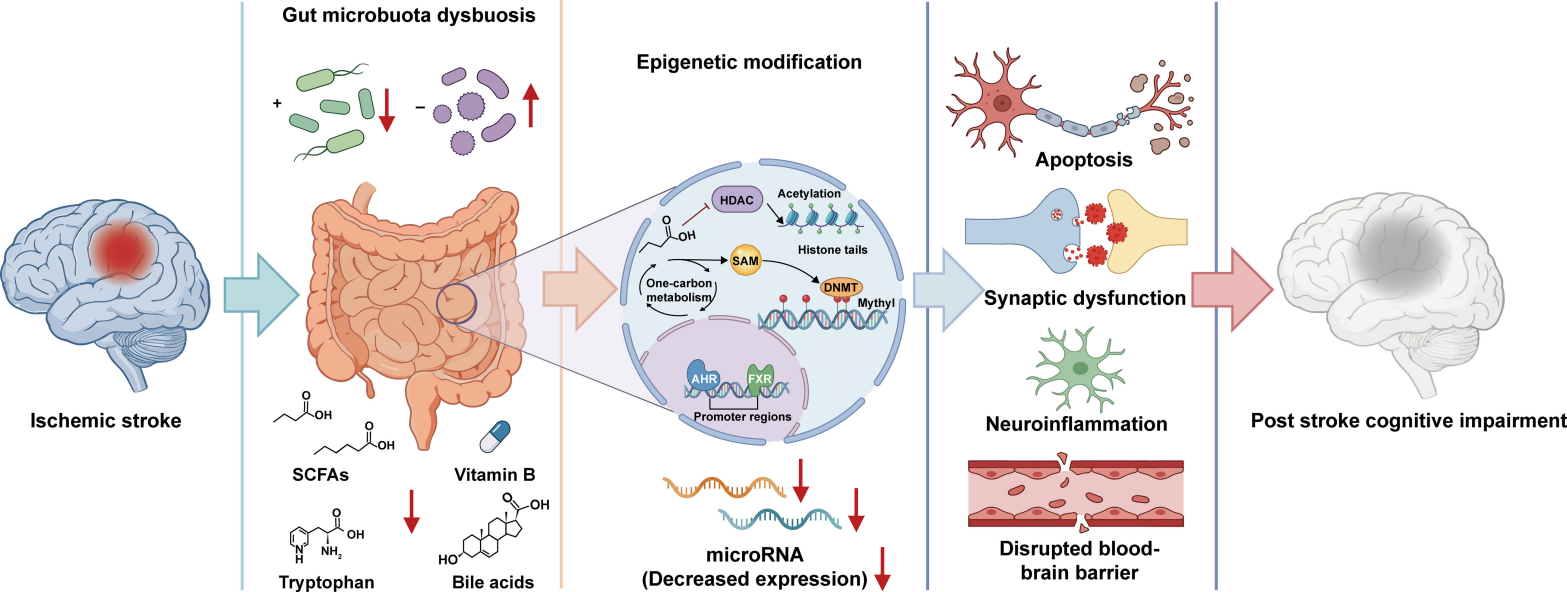
The biological pathway from ischemic stroke to post-stroke cognitive impairment (PSCI). Through gut microbiota dysbiosis, epigenetic modifications, and molecular mechanisms. The diagram illustrates how an ischemic stroke (Left) induces gut microbiota dysbiosis (Middle-Left), leading to altered levels of microbial metabolites such as short-chain fatty acids (SCFAs), tryptophan derivatives, bile acids, and vitamin (B) These metabolites can cross the blood-brain barrier and enter central nervous system (CNS) pathways. In the cell nucleus (Center), the metabolites mediate epigenetic modifications, including histone acetylation (via HDAC inhibition), DNA methylation (via SAM-mediated DNMT activity), and non-coding RNA expression (e.g., microRNAs). These changes regulate transcription at promoter regions influenced by receptors like AHR and FXR. Epigenetic reprogramming affects neuronal apoptosis, synaptic dysfunction and amyloid-beta accumulation, neuroinflammation (activated microglia), and disrupted blood-brain barrier (BBB) integrity (Middle-Right). Collectively, these pathological changes result in PSCI, symbolized by a faded brain icon with impaired cognitive function (Right).

In addition, vitamin B12 is highly demanded by the nervous system, and its deficiency can lead to various neurological manifestations such as cognitive impairment. Experimental data suggest that methylation of m6A in mRNA may contribute to the neurological consequences associated with vitamin B12 deficiency (Mosca et al., 2021). Methylated epigenetic modifications may be a potential mechanism for the action of vitamin B on PSCI. Future studies should deeply resolve the methylation pathways mediated by vitamin B, further exploring the specific molecular pathways and action nodes of different vitamin B isoforms in one-carbon metabolism and methylation. It is essential to clarify how they precisely regulate the methylation levels of DNA, RNA, histones, proteins, and the detailed molecular mechanisms of these regulations in the maintenance of cognitive function and the development of cognitive disorders, and open up a new avenue for elucidating the regulatory functions of the GM and their metabolites in the pathophysiological processes of PSCI. Furthermore, it will provide a theoretical and practical basis for the development of therapeutic regimens, assessment of efficacy and investigation of the mechanism of action of GM and its metabolites in the treatment of PSCI. Major GM metabolites in PSCI (such as SCFAs and vitamin B)-Epigenetic modification mechanisms as shown in **Figure 1**.

### 4.3 The TMAO and neurotransmitters: emerging roles of gut microbial metabolites in epigenetic regulation and post-stroke cognitive impairment

GM utilize choline to produce trimethylamine (TMA), which is further oxidized in the host liver to form trimethylamine-N-oxide (TMAO) (Liu and Tang, 2019). TMAO accelerates brain aging and cognitive decline in mice by promoting neuronal senescence, damaging synapses, and downregulating the expression of proteins associated with synaptic plasticity (Fu et al., 2024). Certain gut microbiota, such as the Firmicutes and Proteobacteria phyla, are capable of producing TMA. The detection rate of these bacterial strains is higher in patients with PSCI, suggesting that changes in the gray matter (GM) of PSCI patients may influence TMAO levels by regulating TMA synthesis in the gut. A growing body of research (Zhu et al., 2020; Gong et al., 2021a) indicates that TMAO is associated with PSCI, with significantly elevated plasma TMAO levels observed in IS patients with cognitive impairment. Neuroinflammation and oxidative stress are key mechanisms by which TMAO induces cognitive deficits; elevated TMAO levels exacerbate neuroinflammation in the hippocampal CA3 region, whereas inhibiting TMAO synthesis can mitigate neuronal damage and neuroinflammation (Shi et al., 2025) A recent study has found that TMAO can directly interfere with the methionine cycle, thereby reshaping the epigenetic landscape. The specific mechanism of action involves TMAO acting as a non-competitive inhibitor within cells, targeting S-adenosylhomocysteine hydrolase (SAHH). This inhibitory effect leads to the intracellular accumulation of its substrate, S-adenosylhomocysteine (SAH). SAH is a key inhibitor of methyltransferases in the body; its accumulation reduces overall methylation capacity (i.e., a decrease in the SAM/SAH ratio), ultimately affecting post-translational modifications of histones, including methylation and acetylation levels. Notably, compared to the liver, the cerebral cortex and hippocampus are more sensitive to these TMAO-induced epigenetic changes, which are directly linked to core brain regions involved in learning and memory (Han et al., 2025). TMAO can also extensively remodel chromatin in endothelial cells by upregulating H3K4me3 and other H3 histone methylation (Matacchione et al., 2024). In summary, TMAO directly damages neurons by inducing neuroinflammation and oxidative stress, and penetrates deep into the cell nucleus. On one hand, it disrupts the methionine cycle by inhibiting SAHH, thereby reshaping the epigenetic landscape of the hippocampus and cortex; on the other hand, it upregulates histone methylation levels, such as H3K4me3, in endothelial cells, extensively mediating chromatin remodeling. These multi-level pathological effects collectively reveal TMAO’s central role in the development of post-stroke cognitive impairment, providing a novel perspective for elucidating its molecular mechanisms.

Besides TMAO, GM can synthesize and regulate various neurotransmitters such as serotonin (5-HT), dopamine (DA), gamma-aminobutyric acid (GABA), and glutamate (Glu), which influence CNS functions by modulating neural transmission processes (Wang et al., 2025). Although only 5% of 5-HT in the human body originates from the brain, approximately 95% is produced in the gastrointestinal tract, primarily by enterochromaffin cells. Certain bacterial strains, such as Lactobacillus and Escherichia coli, directly synthesize 5-HT, though they represent a minority (Qu et al., 2024). Furthermore, GM-mediated dopamine production does not directly enter the brain but involves indirect pathways, including the metabolism of dopamine precursors such as tyrosine, which can cross the blood-brain barrier and be utilized by neuronal cells to synthesize dopamine (Zhang et al., 2023). Given this background, this review focuses on the potential impact of GABA and Glu on the epigenetic mechanisms underlying PSCI.

Evidence from mouse models of stroke has linked elevated GABA levels in the ipsilateral hippocampus with memory deficits. GABA exerts dual physiological roles: serving as an excitatory signal that promotes neurogenesis in immature neurons, while maintaining network homeostasis as an inhibitory signal in mature neurons, which is essential for learning and memory. However, in the context of stroke, sustained GABAergic transmission disrupts this balance, suppressing neurogenesis and long-term potentiation (LTP), ultimately leading to cognitive impairment (Torres-López et al., 2023). The underlying mechanisms include excitotoxicity, neuroinflammation, and the involvement of circulating factors. From a therapeutic perspective, targeting GABAergic pathways has shown promise. For instance, inhibiting the α5-GABAA receptor provides sustained improvement in vascular cognitive impairment (Gacsályi et al., 2018). Additionally, S-ketamine mitigates post-stroke depressive-like behaviors and cognitive deficits by reducing astrocyte proliferation, excessive GABA production, and pro-inflammatory cytokines (Zhang et al., 2023). The interactions between GABAergic signaling and BDNF epigenetic regulation are particularly noteworthy. In models of perioperative neurocognitive disorders, sustained activation of GABAB receptors leads to hyperphosphorylation of downstream signaling proteins, exacerbating Bdnf gene methylation and reducing hippocampal BDNF expression in aged mice (Wu et al., 2025).

Glutamate, the primary excitatory neurotransmitter in the CNS, represents the molecular foundation of learning and memory processes. Under physiological conditions, Glu release and clearance maintain a dynamic equilibrium. However, in neurodegenerative diseases and acute brain injuries, Glu homeostasis is disrupted, leading to excitotoxicity and neuronal death, ultimately contributing to brain atrophy. After ischemic stroke, excessive Glu accumulation in extracellular spaces overactivates NMDAR and AMPAR, triggering excitotoxic cascades that impair cognitive function (Yin, 2024). The gut-brain axis appears to participate in this pathological process. For example, synergistic interaction between D-galactose and L-Glu exacerbates cognitive impairment by increasing amyloid-beta (Aβ1-42) deposition in the cerebral cortex and gut, reducing microbial diversity, damaging the intestinal barrier, and activating inflammatory pathways (Wang et al., 2023). On the other hand, insufficient Glu levels also impair cognitive performance. In models of postoperative cognitive dysfunction, hippocampal CA1 Gluergic neuronal activity, regulated by the SIRT1/BDNF signaling axis, is significantly diminished. Enhancing this pathway restores synaptic plasticity and alleviates cognitive deficits (Wu et al., 2024). Moreover, Glu has garnered increasing attention for its role in epigenetic modulation. Glutamine metabolism, by maintaining elevated α-ketoglutarate (αKG) levels and improving the αKG-to-succinate ratio, facilitates histone and DNA demethylation, ensuring cellular pluripotency (Carey et al., 2015). A similar mechanism has been observed in immune regulation, where extracellular vesicles derived from M2 macrophages deliver Glu, activating αKG/Jmjd3-dependent epigenetic reprogramming in osteoclast precursors (Huang et al., 2024). In tumor cells, Glu metabolism via the NMDAR-Ca²^+^-CaMKII pathway activates RNA methyltransferase METTL3, enhancing HK2 expression through m6A modification and subsequently reprogramming cellular metabolism (Li et al., 2023). In epilepsy models, Glu-induced neuronal hyperexcitability triggers a progression from transient histone modifications to persistent DNA methylation, resulting in hypermethylation of the Gria2 and Grin2a promoter regions and the establishment of a pro-epileptic “cellular memory” (Kiese et al., 2017). However, direct evidence linking Glu and GABA to epigenetic pathways in PSCI remains elusive and warrants further investigation.

The gut microbiota regulate CNS function by modulating the synthesis, metabolism, and activity of neurotransmitters such as 5-HT, GABA, and Glu. In PSCI, excessive activation of the GABAergic system and Glu imbalance jointly underlie the neurochemical basis of cognitive impairment. Existing evidence suggests that gut microbiota not only indirectly influence the synthesis and release of neurotransmitters in the brain via metabolites (e.g., SCFAs) but also shape precursor metabolism, thereby modulating post-stroke neural functions within the gut-brain axis framework. Future research should focus on elucidating the intricate interplay between gut microbiota, neurotransmitter metabolism, and central epigenetic mechanisms. Such findings will provide a robust theoretical foundation for the development of PSCI interventions targeting the gut-brain axis.

### 4.4 GM-derived metabolites in PSCI: mechanistic pathways and clinical implications

#### 4.4.1 TRP metabolites and aryl hydrocarbon receptor signaling

TRP is an essential amino acid, and TRP catabolic pathways may contribute to neurodegenerative diseases like AD (Choe et al., 2024). GM regulates the three major TRP metabolic pathways to produce serotonin (5-hydroxytryptamine, 5-HT), kynurenine (Kyn) and indole derivatives (Agus et al., 2018). Among these metabolites, indole derivatives and Kyn exert pivotal functions in maintaining intestinal homeostasis and systemic immunity, and may additionally contribute significantly to the initiation and progression of neurological disorders, cardiovascular disease, and liver fibrosis (Su et al., 2022). Reduced TRP uptake leads to a decrease in 5-HT, which impedes vagal activity, leading to hippocampal responses and memory impairment (Wong et al., 2023). It is noteworthy that these metabolites can exert diverse effects by activating or inhibiting aryl hydrocarbon receptors (AHRs). Activated AHRs stimulate the expression of postsynaptic proteins and promote hippocampal development (Gao et al., 2020). The level of AHR nuclear transporter protein 2 in the hippocampus is potentially linked to cognitive-behavioural functions in rats with post-stroke depression (Pei et al., 2019). Furthermore, AHRs agonists can selectively modulate the epigenetic profiles of target genes driven by exogenous response elements, thereby affecting transcriptional responses (Joshi et al., 2017).

However, Rzemieniec found that blocking the AHR signalling pathway may be a promising strategy in stroke therapy. This is because AHR inhibitors reduce neuronal apoptosis, infarct size and oedema progression and protect hippocampal neurons from hypoxia/ischemia (Rzemieniec et al., 2021). These two opposing conclusions may arise because of the degree of control over confounding factors or different signaling molecules targeted and need to be explored further. Importantly, the signaling pathways activated by AHRs are closely related to epigenetic modifications, and activation of AHRs also reduces DNMT expression levels (Wajda et al., 2020). AHRs regulate chromatin remodeling by controlling histone acetylation, methylation, miRNAs and also long non-coding RNAs, but the pathways are unclear (Disner et al., 2021). Studies have shown that AHRs can promote tumorigenesis by targeting HDAC expression and altering epigenetic regulation. However, whether this process occurs in PSCI is unknown, but by observing that activation or inhibition of AHRs influences pathophysiological changes after stroke onset, we learned that TRP metabolism and its interactions with AHR signaling pathways are important in the pathogenesis of PSCI. In-depth investigation of these mechanisms, especially the role of AHRs in cognitive function after stroke, is expected to provide new targets and strategies for the prevention and treatment of PSCI. Future studies should focus on the following directions: the effect of GM on TRP metabolism and AHR activity; the interaction between TRP metabolism and the AHR pathway; the mechanism of AHR-mediated epigenetic regulation; and the development of individualized therapeutic strategies based on the GM-TRP-AHR pathway. These studies will not only help elucidate the pathogenesis of PSCI, but may also provide new ideas for the treatment of other neurodegenerative diseases such as AD.

#### 4.4.2 Bile acids and farnesol X receptor-mediated epigenetic regulation

GM can also synthesize over 20 different metabolites from primary bile acids of host origin via microbial hydroxysteroid dehydrogenase (Winston and Theriot, 2020). Bile acids have been shown to play a significant role in the CNS (Lirong et al., 2022), participating in neuroinflammatory and Amyloid β-protein(Aβ) processes in the brain. For example, Weng (Weng et al., 2024) demonstrated that GM imbalance leads to abnormal bile acid metabolism thereby promoting neuroinflammation and dysfunction, and they found that lithocholic acid triggers peripheral and brain inflammation, disrupts the BBB, and establishes a vicious cycle leading to a sustained inflammatory response. Similarly, Zhou’s study utilized metabolomics to target bile acids, found that AD model mice had elevated levels of certain primary bile acids and secondary bile acids including lithocholic acid, foiiowing treatment with Alpiniae oxyphyllae, may improve learning and memory deficits in AD mice by modulating bile acid metabolism, and alleviate Aβ deposition in the brains of model mice (Zhou et al., 2022). It is known that the conversion of bile acids to unbound forms by the GM can activate the farnesol X receptor (FXR) (Jia et al., 2018; Cheung et al., 2023). Activated FXR regulates the expression of certain genes through epigenetic modifications. For example, FXR activation promotes E2F3 expression and β-cell proliferation by enhancing the recruitment of the histone acetyltransferase steroid receptor coactivator-1 (SRC1) to the promoter region of the E2f3 gene in diabetic rats (Kong et al., 2024). However, there are few studies on the regulation of epigenetic modifications by bile acids, and whether bile acids are involved in epigenetic modifications through the activation of FXRs need to further research, thereby regulating the expression of genes related to cognitive function. Future studies can use ChIP-seq, RNA-seq and other techniques to analyse the changes in epigenetic modification of target genes after FXR activation; screen key genes related to cognitive function and verify their role in PSCI.

## 5 Conclusion and outlook

This article reviews recent advances in the regulation of PSCI by GM and its metabolites through epigenetic mechanisms.GM dysregulation and altered metabolite production play an important role in the development and progression of PSCI by affecting DNA methylation, histone modification and non-coding RNA regulation. Stroke, as the leading cause of death and disability worldwide, and its subsequent triggering of cognitive impairment cannot be ignored. However, due to the prolonged pathogenesis and difficult diagnosis of PSCI, this not only seriously affects the quality of life of patients but also imposes a heavy economic burden. Despite the enormous social and economic impact of PSCI, the current understanding of its etiology and pathogenesis is still very limited, and there is a lack of effective cures or specific treatments. Therefore, it is particularly important to actively explore new therapeutic targets for PSCI. Early studies have found that GM plays a unique role in CNS by communicating with the brain through the ‘MGB axis’, which in turn affects brain function and behavior. This process involves multiple signaling pathways, including the vagus nerve, endocrine and immune systems, and can cause a wide range of physiopathological changes. GM imbalance exacerbates BBB destruction, inducing systemic inflammation and neuroinflammation, leading to neurodegenerative pathologies. It has been shown that the structure of GM is disturbed after stroke onset, which further confirms the close association between GM and PSCI. In recent years, with the in-depth study of PSCI, the unique changes of GM in PSCI and its potential role have been gradually recognized. More critically, GM plays a central regulatory role in the development of PSCI through its metabolites such as SCFAs, vitamin B, etc. These metabolites can regulate epigenetic modification processes that are closely linked to gene expression, including DNA methylation, histone modification and regulation of non-coding RNAs, thereby altering cellular function and phenotype. These epigenetic modifications have profound impacts on organismal growth and development, cell differentiation, disease development and environmental adaptation. For instance, it has been shown that transplantation of SCFA-enriched fecal microbiota into IS model rats significantly increased their butyrate levels, which in turn effectively ameliorated PSCI symptoms. This effect is closely related to the inhibitory effect of butyrate on HDAC during histone modification. Moreover, SCFAs can regulate host non-coding RNA levels, exert anti-inflammatory effects, promote the restoration of intestinal barrier function, and reduce the transmission of inflammatory factors to the central nervous system via the ‘gut-brain axis’, thereby reducing the neuroinflammatory response. Simultaneously, B vitamins also play an indispensable role in maintaining neural function in the brain. They are involved in the one-carbon pathway, which directly influences the methylation status of DNA, RNA, histones and proteins. Other genetically engineered metabolites, such as TRP metabolites and bile acids, can also regulate the epigenetic modification process in the host, thus influencing the pathogenesis of PSCI.

However, most of the current studies on GM and PSCI are based on animal models, and cases of direct treatment of clinical PSCI patients using probiotics have not been reported with limited clinical data. Due to the difficulty of isolation and cultivation of specific strains, the means of faecal transplantation used in therapeutic animal models are mostly with mixed strains, which cannot clarify the effective strains in different diseases. Moreover, the presence of abundant microbial species, genetic composition, and heterogeneity in spatial and temporal distribution in the gut results in high GM heterogeneity and difficulty in standardization. Future studies can integrate multi-omics, such as macro-genome-based combined with single-bacteria transcriptome and epigenome to detect and precisely regulate the GM of clinical PSCI patients, and implement personalised treatment protocols for different individuals based on the test results. As there are also transcriptional differences in some strains at different time points and heterogeneity in the metabolic activities of different strains, the timing of GM intervention can be optimised in the future. It is also possible to integrate longitudinal cohort studies based on multi-omics analysis technology to observe the evolution of the entire disease process, obtain multi-dimensional molecular and pathway information of biological samples, and enhance the effectiveness and efficiency of disease research, target finding and marker discovery. These methods provide brand new tools for precision microbiome diagnosis and targeted flora intervention, and provide new therapeutic means for PSCI, to further develop microbiome therapy or supplementation of related metabolites, contributing to the improvement of human health. Although the GM composition of PSCI patients has not been found to be identical in different studies, as a whole, PSCI patients have a reduced abundance of beneficial bacteria and an increased number of conditionally pathogenic bacteria in their organisms. This further leads to changes in GM metabolites, especially SCFAs, among which sodium butyrate, as an important epigenetic regulatory molecule, exerts a significant effect on the treatment of PSCI. Animal studies have now demonstrated that sodium butyrate supplementation in PSCI models can alleviate cognitive impairment by affecting epigenetic, which is of great significance for the treatment of clinical PSCI. Subsequent studies are needed to explore the epigenetic modifications of SCFAs or other metabolites that exert beneficial effects on PSCI through a large number of studies, which will provide potential possibilities for future studies on the mechanisms of cognitive rehabilitation in PSCI patients. Another important consideration for future research and clinical translation is the temporal heterogeneity of PSCI. Preventive and therapeutic strategies targeting gut microbiota and epigenetic regulation should not be uniform across all stages after stroke. Instead, stage-specific interventions may be required for the acute, subacute, and chronic phases post-stroke. In the acute phase, strategies may focus on reducing neuroinflammation, stabilizing the blood–brain barrier, and preventing neuronal apoptosis, potentially through modulation of gut microbiota composition and anti-inflammatory metabolites such as short-chain fatty acids. During the subacute phase, therapeutic approaches may aim to promote neuroplasticity, synaptic remodeling, and neuronal survival, possibly through epigenetic regulation of neurotrophic factors such as BDNF and NGF. In the chronic phase, interventions may focus on long-term cognitive rehabilitation, modulation of chronic neuroinflammation, and maintenance of microbiota homeostasis. Therefore, future studies should consider the dynamic changes in gut microbiota, microbial metabolites, and epigenetic modifications across different post-stroke stages and develop time-specific intervention strategies for PSCI prevention and treatment.

We propose a new regulatory network model that reveals the key molecular mechanisms by which GM and its metabolites affect PSCI through DNA methylation, histone modification and non-coding RNA regulation by integrating the latest findings from microbiomics, epigenetics and neuroscience, providing new ideas for multidisciplinary cross-sectional studies and a theoretical framework for future research. The study of epigenetic mechanisms in the development of PSCI provides new perspectives for understanding the complexity of the pathogenesis of PSCI and offers the possibility of developing new preventive and therapeutic means. Future studies should further explore the specific molecular mechanisms between GM and epigenetic regulation and develop diagnostic and therapeutic approaches for PSCI based on GM and its metabolites. In addition, the development of individualized therapeutic strategies must consider the GM composition and epigenetic characteristics of patients. We believe that further studies will provide new insights into the complex topic of the role of epigenetics in the ‘MGB axis’.
